# Parent–Infant Attachment Insecurity and Emotional Eating in Adolescence: Mediation through Emotion Suppression and Alexithymia

**DOI:** 10.3390/nu13051662

**Published:** 2021-05-14

**Authors:** Roseriet Beijers, Marta Miragall, Yvonne van den Berg, Hanna Konttinen, Tatjana van Strien

**Affiliations:** 1 Behavioural Science Institute, Radboud University, 6525 XZ Nijmegen, The Netherlands; y.vandenberg@psych.ru.nl (Y.v.d.B.); t.vanstrien@psych.ru.nl (T.v.S.); 2 Donders Institute, Radboudumc, 6525 GA Nijmegen, The Netherlands; 3 Department of Personality, Evaluation, and Psychological Treatments, University of Valencia, 46010 València, Spain; marta.miragall@uv.es; 4 CIBER Fisiopatología Obesidad y Nutrición (CIBEROBN), Instituto Carlos III, 28029 Madrid, Spain; 5 Faculty of Social Sciences, University of Helsinki, 00100 Helsinki, Finland; hanna.konttinen@helsinki.fi

**Keywords:** parent–child attachment, strange situation procedure, attachment Q-set, emotional eating, emotion suppression, alexithymia

## Abstract

Emotional eating (EE), the propensity to eat in response to emotions, is thought to have its origins in the early parent–infant relationship. This study tested the hypothesis that infant attachment insecurity results in EE in adolescence through the increased use of the emotion regulation strategy suppression of emotions and subsequent alexithymia. At the age of 15 months, parent–infant attachment security (*n* = 129) was observed with two abbreviated attachment measures: the shortened strange situation procedure (SSSP), and the shortened attachment Q-set (S-AQS). At the age of 12 years, children completed self-report questionnaires to assess the suppression of emotions, alexithymia, and EE. At the age of 16 years, EE was measured again. The mediation models indicated that lower parent–infant attachment security predicted increased use of suppression of emotions, which was related to increased alexithymia, and in turn more EE at the age of 12 years. These results were similar and significant for both attachment measures, and also (marginal) significant with EE at the age of 16 years as an outcome. Lastly, when parental caregiving quality was included, the models with the SSSP as predictor remained significant, but the models with the S-AQS became insignificant. These results indicated that to a certain extent, infant attachment security could predict adolescent EE above and beyond parental caregiving quality.

## 1. Introduction

Emotional Eating [EE], the propensity to eat in response to emotions, is considered an atypical response to stress. As the physiological stress response mimics the internal sensations associated with feeding induced satiety, the typical response to stress is not eating [1]. When stressed, emotional eaters tend to eat energy-dense food (e.g., [2,3,4,5]), which in turn can result in weight gain and obesity [6,7,8]. It is therefore important to increase our understanding of the antecedents of EE.

### 1.1. Parent-Infant Attachment and EE

EE is thought to have its origins in the early caregiving environment [9,10], including the parent–infant attachment relationship. Attachment is the emotional bond that forms between the infant and the primary caregiver(s), usually the parent(s) [11]. In times of stress, securely attached infants go to the parent for protection and comfort, after which they return to exploring the world around them. Insecurely attached infants are not able to use the parent as a “safe haven” or “secure base”, and experience difficulties coping with stress. In this light, longitudinal research has shown that infant attachment insecurity forecasts a range of later negative child developmental outcomes, including socio-emotional behavior [12]. Though EE is considered an atypical response to stress, and as such might represent difficulties coping with stress, it is yet unclear whether infant’s attachment insecurity also forecasts later EE.

Though associations between attachment insecurity and unhealthy eating behaviors have been consistently found [13,14,15], the studies to date mostly focused on adult attachment representations, using self-report, and employed cross-sectional designs. Both adult attachment representations and EE stem from complex developmental processes with their onset in infancy. Longitudinal studies starting in early life are thus clearly needed to start unravelling cause–effect associations between attachment and EE [13,16]. Moreover, previous studies on attachment insecurity and unhealthy eating behaviors mostly focused on eating pathology in clinical samples. For example, higher rates of insecure attachment have been found in patients with eating disorders, including anorexia nervosa and bulimia nervosa, compared to rates in community samples [17,18,19]. By focusing on pathological eaters, it remains unclear whether attachment insecurity is linked to eating behaviors in the general population [13]. The few studies that did investigate attachment security and EE, indicated that attachment insecurity increases the risk for EE in bariatric surgery candidates and patients [20,21,22], and that EE mediated the association between attachment anxiety and body mass index (BMI) [23], but not between disorganized and BMI [15]. As such, the first aim was to longitudinally investigate if attachment security observed in infancy predicts EE in adolescence.

### 1.2. Measures of Parent-Infant Attachment

We measured parent-infant attachment security with two abbreviated measures of well-known attachment measures: The strange situation procedure (SSP) [24], and the attachment Q-set (AQS) [25,26]. The SSP and the AQS are different in many ways. The SSP is a structured procedure at the laboratory to assess whether infants can use the parent as a safe haven to return to in times of stress [24]. The abbreviated version of the SSP uses one separation, instead of two, to observe the child’s reunion behavior towards the parent. The AQS is an observation at home to assess whether infants can use the parent as a secure base. The AQS consists of 90 cards describing attachment-related behaviors. The observer sorts the cards and a security score is obtained by correlating the child’s individual sort with the sort of a prototypically secure child [25,26]. The abbreviated version of the AQS implies that the observation was based on 2 h, instead of 3X3 h, of home observations. Both abbreviated measures of the SSP and the AQS have been shown valid [27,28,29].

The SSP and the AQS measures reveal how the child’s attachment system functions in different situations. The SSP focuses on the dynamics of the attachment system in new, stressful situations, while the AQS focuses on the dynamics in the natural home setting [30]. In this light, this study will exploratorily examine whether both attachment measures can predict adolescent EE.

### 1.3. Mediators between Parent-Infant Attachment and EE

Accumulating evidence suggests that dysfunctional emotion regulatory capacities serve as a potential underlying mechanism relating attachment insecurity to EE [22,31,32,33]. Securely attached infants are better able to regulate and modulate their emotions through the process of co-regulation [34,35,36,37,38,39]. Co-regulation refers to the process of responsive parents protecting the infant from high levels of stress, and simultaneously promoting the infant’s own emergent capacities to regulate stress. Insecurely attached children are thus suggested to have developed dysfunctional emotion regulatory capacities [34,35,36], which in turn might predict EE.

Increased use of emotion suppression is suggested to be such a dysfunctional emotion regulation strategy [40]. Suppression of emotions involves the inhibition of expression of ongoing emotions and related behaviors. While lower parenting quality has been associated with an increased use of suppression of emotions [40,41], we are not aware of studies linking attachment insecurity to this emotion regulation strategy. However, one study in mothers and their 4,5-year-old children found attachment insecurity to be associated with higher child avoidance to discuss emotions of sadness and anger [42]. This study provides preliminary evidence that attachment insecurity is associated with suppression of emotions. In turn, suppression of emotions has been associated with alexithymia [40,43,44]. Alexithymia is defined as the inability to identify and describe emotions experienced by one’s self or others. As studies have related alexithymia to EE [40,45,46], the second aim was to investigate whether the emotion regulation strategy of emotion suppression, and subsequent alexithymia, mediates the infant attachment insecurity-adolescent EE association.

### 1.4. Aims of the Present Study

We aimed to test the hypothesis that parent-infant attachment insecurity predicts increased use of suppression of emotions, which relates to alexithymia, and subsequent EE in adolescence. In an earlier study on the same cohort, lower parental caregiving quality in infancy forecasted more EE in adolescence [40]. On this basis, and the notion that sensitivity is one, but not only antecedent of attachment security [47], we also investigated the hypothesis that infant attachment security can predict adolescent EE, above and beyond parental caregiving quality. By identifying possible predictors and developmental pathways by which the vulnerability to develop EE across development increases, this study contributes to assessment, prevention and treatment of EE.

## 2. Method

### 2.1. Participants

The data came from the ongoing Nijmegen longitudinal study [NLS]. Families with a 15-month-old infant were recruited via local health-care centers in Nijmegen (The Netherlands). Inclusion criteria were having sufficient fluency in Dutch, and having a child without serious health conditions. Of the 639 families approached, 174 were interested in participation and 129 families were randomly selected (the maximum number given the time and resources available for the study). For more information on the NLS, see [48,49].

**Figure 1:**
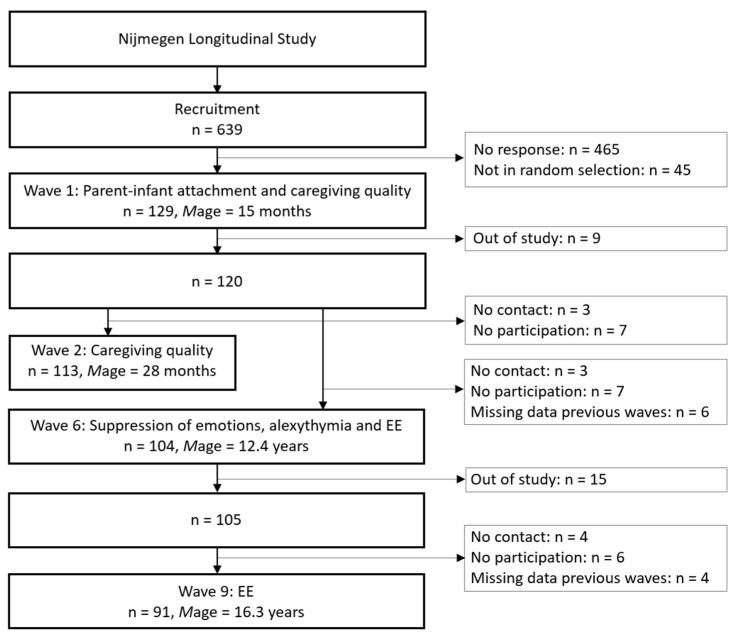
Nijmegen Longitudinal Study (NLS) flowchart.

The 129 children (67 boys, 62 girls) had a mean age of 15.1 months old (*SD* = 0.3 months). The majority of the children were from two-parent families (95%) and from families in which the mother was the primary caregiver (98%). The primary caregivers were between 22 and 47 years old (*M* = 32.9 years, *SD* = 4.4) and representative of the Dutch population in socioeconomic background. For the present study, 104 children had complete data until the age of 12 years. When the EE measure at the age of 16 years was included, 91 children had complete data. See also Figure 1 for the NLS flowchart. The study procedures were approved by the Institutional Review Board of the Radboud University (ECG-20213-1811-157).

### 2.2. Procedure

At the age of 15 months, the two abbreviated measures to measure infant attachment security were performed during a visit to the laboratory and a home visit. Additionally, a 12 to 15 min semi-structured parent-infant interaction was recorded during the home visit. During the 28-month assessment, another parent–child interaction was recorded during a home visit. At the age of 12 years, data were collected during a home visit. Children completed questionnaires to assess their use of suppression of emotions, alexithymia, and EE. At the age of age 16 years, data were collected during a school visit. Children completed a questionnaire on EE.

### 2.3. Measures

#### 2.3.1. The Shortened Strange Situation Procedure (SSSP)

The Shortened Strange Situation Procedure (SSSP) consists of three episodes: parent and infant in the room (3 min), infant alone (4 min), and parent–infant reunion (3 min) [24]. To increase the stressfulness, the duration of the separation was increased from 3 min to 4 min. Two trained coders rated the videotaped SSSP’s and classified the infants as secure (B), avoidant (A), resistant (C), or disorganized (D). With 95% agreement on the main classifications, intercoder reliability was adequate (*n* = 20 cases). More information regarding the validity of the SSSP and its scoring, see [30,49]. For this study, a dummy variable was created representing secure attachment (B) versus insecure attachment (A, C, and D). In our sample of 104 participants at the age of 12 years, 67 infants (64%) had been classified as securely attached and 37 infants (36%) as insecurely attached (i.e., 16 infants with label A, 8 infants with label C, and 13 infants with label D).

#### 2.3.2. The Shortened Version of the Attachment Q-Set (S-AQS)

The Dutch translation of the AQS [25] was applied on two hours of home observations, which is considerably less than the 3X3 h prescribed by Waters and Deane [26]. While larger effect sizes are indicated for the association between SSP security and AQS security when AQS observations lasted >three hours, compared to AQS observations of ≤three hours, the AQS observation duration did not affect the association between AQS security and later child outcomes [29]. The observer was trained until a reliability of 0.75 was reached. Subsequent reliability checks for five independent sorts for the same children exceeded the standard of 0.75. More information regarding the validity of the S-AQS and its scoring, see [30,49].

#### 2.3.3. Emotion Regulation Strategy of Suppression of Emotions (12 Years)

Children completed the 10-item Emotion Regulation Questionnaire [50]. Four items measure suppression of emotions. The items were rated along a seven-point scale ranging from strongly disagree to strongly agree. Higher scores reflect more use of suppression of emotions.

#### 2.3.4. Alexithymia (12 Years)

Alexithymia difficulty identifying feelings was measured with the corresponding subscale of the Toronto Alexithymia Scale-20 (TAS-20) [51,52,53]. The subscale has seven items that were rated along a three-point scale. Higher scores reflect more alexithymia.

#### 2.3.5. Emotional Eating (EE; 12 Years and 16 Years)

EE at the age of 12 years was assessed with seven items of an age-adapted 20-item version of the Dutch Eating Behavior Questionnaire (DEBQ), which is suitable for 7- to 12-year-old children (DEBQ-C [54]). The items were rated along a 5-point scale ranging from never to very often. Higher scores reflect more EE.

EE at the age of 16 years was assessed with six items of the brief version of the adult version of the DEBQ [55]. The items were rated on a five-point scale from never to very often. Higher scores reflect more EE.

#### 2.3.6. Parental Educational Level

Maternal and paternal educational level were rated on a 7-point scale. Because of high intercorrelations (*r* = 0.69), the mean of paternal and maternal educational level was calculated and included as confounder in the main analyses.

#### 2.3.7. Parental Caregiving Quality (15 and 28 Months of Age)

To test whether infant attachment security can predict adolescent EE, above and beyond parental caregiving quality [40], parental caregiving quality was observed at child age 15 and 28 months. The two recorded parent–child interactions were rated for: (1) supportive presence (e.g., the extent to which caregivers provide emotional support); (2) respect for the autonomy of the child (e.g., the extent to which caregivers adjust to their child and do not interfere with ongoing activity); and (3) hostility (i.e., the extent to which caregivers express anger or rejection of the child). Independent coding of 25 cases yielded interrater reliabilities above 0.83 for all scales. Parental caregiving quality was computed with the SOM ratio of positive parenting to the sum of positive plus negative parenting. For further details, see [40].

### 2.4. Data Analyses

Descriptive statistics and Pearson’s correlation between the study variables were conducted in order to analyze the mean, standard deviation, and relationships between the study variables. These preliminary analyses were conducted both within the sample of completers of the DEBQ-C at the age of 12 years, and within the sample of completers of the DEBQ at the age of 16 years.

For the main analyses, serial multiple mediator analyses were performed with the macro PROCESS version 2.16 for SPSS [56]. Model 6 was chosen to test whether the association between attachment security and EE was mediated by suppression of emotions and alexithymia. Four mediation analyses were conducted as the models were separately tested for the two variables of attachment security (SSSP and S-AQS), and separately tested for completers of the eating behavior questionnaire at the age of 12 and 16 years. Child sex and parental educational level were entered as covariates. The mediational effect was significant, respectively borderline significant, when the 95%, respectively, 90% confidence intervals (CIs) of the indirect effect did not include the zero-value. Regression coefficients are reported in unstandardized form as b-values.

Subsequently, the confidence intervals of the three specific indirect effects were inspected: (1) the single mediation effect through emotion suppression, (2) the single mediation effect through alexithymia difficulty identifying emotions, and (3) the serial mediation effect through emotion suppression and alexithymia. Bias-corrected bootstrap 95% CI based on 5000 samples were used. The bootstrapping approach has been recommended by several authors to test mediation. Compared to more traditional methods, the bootstrapping approach has the highest statistical power and the best Type I error control yielding results that are more accurate and less affected by sample size [57,58,59].

As we found in an earlier study on the same cohort that lower parental caregiving quality in infancy forecasted more EE in adolescence [40], all models will be re-run including parental caregiving quality to investigate whether infant attachment security can predict adolescent EE, above and beyond parental caregiving quality.

## 3. Results

### 3.1. Preliminary Analyses

Descriptive statistics and Pearson’s correlations between the study variables are shown in Table 1. The Pearson’s correlations without brackets present the correlations for the group of completers of the DEBQ-C at the age of 12 years (*n* = 104), and the Pearson’s correlations between brackets present the correlations for the group of completers of the DEBQ at the age of 16 years (*n* = 91). No outliers were detected.

#### Correlations

Within the group completers of the DEBQ-C at the age of 12 years, the two attachment security measures (SSSP and S-AQS) were positively correlated, and the strength of the association was moderate. None of the two attachment security measures were significantly associated with EE. Both measures of attachment security were associated with suppression of emotions, indicating that higher attachment security was associated with lower suppression of emotions. More suppression of emotions was moderately associated with more alexithymia difficulty identifying feelings, and more alexithymia difficulty identifying feelings was moderately associated with higher EE.

Within the group completers of the DEBQ at the age of 16 years, attachment security, as assessed with the SSSP, was not associated with EE at the age of 16 years. In contrast, and remarkably, attachment security as measured with the S-AQS was significantly and positively associated with EE, indicating that higher attachment security was associated with higher degrees of EE. A significant negative relationship was found between attachment security as measured by the SSSP and suppression of emotions, whilst the negative relationship between attachment security as measured by the S-AQS and suppression of emotions just failed to reach significance. More suppression of emotions was associated with more difficulty identifying feelings, and more difficulty identifying feelings was associated with higher degrees of EE. Finally, female adolescents and adolescents with more educated parents reported higher degrees of EE at the age of 16 years.

### 3.2. Serial Multiple Mediation Analyses

#### 3.2.1. Emotional Eating at 12 Years

**Figure 2:**
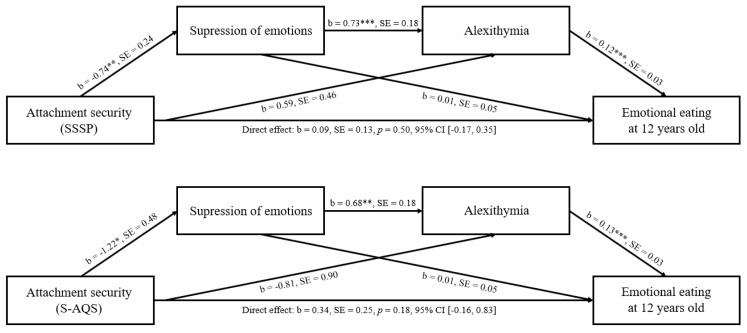
Suppression of emotions and alexithymia as mediators between attachment security and emotional eating at the age of 12 years old. Notes. * *p* < 0.05; ** *p* < 0.01; *** *p* < 0.001. b = unstandardized regression coefficients; SE = standard errors; SSSP = shortened strange situation procedure, S-AQS = shortened attachment Q-set.

Though there was no significant association between infant attachment security, as measured with both the SSSP and S-AQS, and adolescent EE, there is agreement that mediation can exist even in the absence of an overall significant association [60,61]. Therefore, we proceeded to test the mediation models. Unstandardized regression coefficients and standard errors of the full model, and confidence intervals of the direct effects, are shown in Figure 2.

##### Attachment Security as Measured by the SSSP

The total indirect effect (the difference between total and direct effect (c-c’)) was not significant (b = 0.00, SE = 0.08, 95% CI (−0.16, 0.14)). The indirect effects associated with model 1 and model 2 were not significant (b = −0.01, SE = 0.04, 95% CI (−0.11, 0.07), and b = 0.07, SE = 0.06, 95% CI (−0.04, 0.21), respectively). Only the indirect effect of model 3 (the serial mediation effect through emotion suppression and alexithymia) was significant (b = −0.07, SE = 0.03, 95% CI (−0.15, −0.02)). The multiple regression model including all the variables was significant (*F*(5,98) = 5.91, *p* < 0.001), and explained 23.2% of the variance in EE.

##### Attachment Security as Measured by the S-AQS

Again, the total indirect effect was not significant (b = −0.21, SE = 0.15, 95% CI (−0.56, 0.04)). The indirect effects associated with model 1 and model 2 were also not significant (b = −0.01, SE = 0.06, 95% CI (−0.17, 0.10), and b = −0.10, SE = 0.12, 95% CI (−0.38, 0.11), respectively). Only the indirect effect of model 3 (the serial mediation effect through suppression of emotions and alexithymia) was significant (b = −0.10, SE = 0.05, 95% CI (−0.23, −0.02)). The multiple regression model including all the variables was significant (*F*(5,98) = 6.27, *p* < 0.001), and explained 24.25% of the variance in EE.

##### Parental Caregiving

To investigate whether infant attachment security can predict adolescent EE, above and beyond parental caregiving quality, models were re-run including parental caregiving quality. In the model with SSSP attachment security, the indirect effect of model 3 remained significant (b = −0.05, SE = 0.02, 95% CI (−0.12, −0.01)), but when attachment security was measured with the S-AQS, the indirect effect of model 3 became insignificant (b = −0.04, SE = 0.04, 95% CI (−0.13, 0.02)).

#### 3.2.2. Emotional Eating at 16 Years

**Figure 3:**
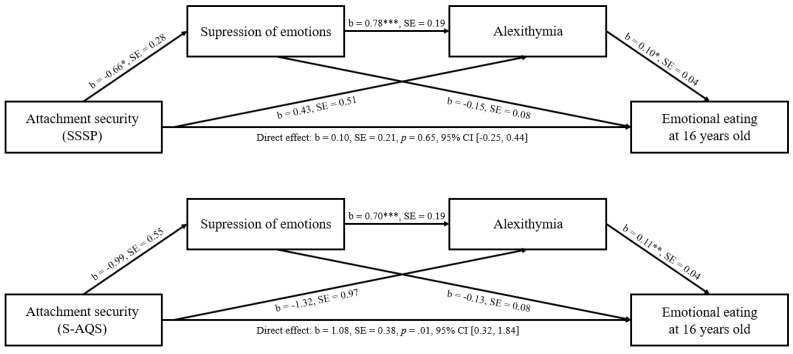
Suppression of emotions and alexithymia as mediators between attachment security and emotional eating at the age of 16 years old. Notes. * *p* < 0.05; ** *p* < 0.01; *** *p* < 0.001. b = unstandardized regression coefficients; SE = standard errors; SSSP = shortened strange situation procedure, S-AQS = shortened attachment Q-set.

The analyses were re-conducted with EE at the age of 16 years. See Figure 3 for the unstandardized regression coefficients and standard errors for the full model, in addition to the confidence intervals of the direct effects.

##### Attachment Security as Measured by the SSSP

The total indirect effect was not significant (b = 0.09, SE = 0.08, 95% CI (−0.04, 0.30)), and the same held true for the indirect effects of model 1 and model 2 (b = 0.10, SE = 0.07, 95% CI (0.01, 0.31) and b = 0.04, SE = 0.07, 95% CI (−0.03, 0.29), respectively). The indirect effect of the serial mediation model 3 was not significant at 95% CI (b = −0.05, SE = 0.04, 95% CI (−0.20, −0.02)), but it was significant at 90% CI (b = −0.05, SE = 0.04, 90% CI (−0.17, −0.01)). The regression model which included all the variables was significant (*F*(5,85) = 7.15, *p* < 0.001), and explained 29.6% of the variance in EE.

##### Attachment Security as Measured by the S-AQS

The total indirect effect was not significant (b = −0.10, SE = 0.16, 95% CI (−0.55, 0.13)). Moreover, the indirect effects associated with model 1 and model 2 were not significant (b = 0.13, SE = 0.12, 95% CI (−0.01, 0.51), and b = −0.15, SE = 0.12, 95% CI (−0.54, 0.00), respectively). The indirect effect of model 3 was significant at 95% CI (b = −0.08, SE = 0.07, 95% CI (−0.32, −0.0006)). The multiple regression model including all the variables was significant (*F*(5,85) = 9.37, *p* < 0.001), and explained 35.5% of the variance in the EE.

##### Parental Caregiving Quality

To investigate whether infant attachment security can predict adolescent EE, above and beyond parental caregiving quality, models were re-run including parental caregiving quality. In the model with SSSP attachment security, the indirect effect of model 3 was significant (b = −0.04, SE = 0.04, 95% CI (−0.19, −0.004)), but when attachment security was measured with S-AQS, the indirect effect of model 3 became insignificant (b = −0.01, SE = 0.05, 95% CI (−0.17, 0.06)).

## 4. Discussion

The present study tested the hypothesis that attachment insecurity in infancy results in emotional eating (EE) in adolescence through the increased use of the emotion regulation strategy of emotion suppression and subsequent alexithymia. We examined whether this hypothesis would hold for the two abbreviated versions of the attachment measures used in this study: The shortened strange situation procedure (SSSP) [24], and the shortened version of the attachment Q-set (S-AQS) [25,26]. In support of our hypotheses, the mediation models indicated that lower parent–infant attachment security in infancy predicted increased suppression of emotions, which in turn was related to more alexithymia, and ultimately more EE at the age of 12 years. These results were similar and significant for both attachment measures. To test the robustness of the chain as a chain in real time, the analyses were reconducted with EE at the age of 16 years. The serial mediation model with EE at the age of 16 years was significant when attachment security was observed with the S-AQS, and marginally significant when attachment security was observed with the SSSP. Lastly, when parental caregiving quality was included, the models with the SSSP as predictor remained significant, but the models with the S-AQS became insignificant. These results indicated that, to a certain extent, infant attachment security can predict adolescent EE above and beyond parental caregiving quality.

Our results suggest that parent–infant attachment insecurity may increase the risk for EE in adolescence through difficulties with emotion regulation and alexithymia. Though associations between attachment insecurity and unhealthy eating behaviors have been consistently found (e.g., [13,14]), the studies to date mostly focused on adult attachment representations and employed cross-sectional designs. The present longitudinal and prospective study thus importantly adds to the field, as longitudinal studies help to unravel cause-effect associations between early attachment and later EE [13,16]. Moreover, this study indicated that not only early parental feeding practices [62,63,64,65], but also a more general quality of the parent–infant interaction was associated with EE in adolescents.

A closer investigation of the results indicated that the direct association between the S-AQS attachment measure and EE at the age of 16 years was significant and positive. Apparently, higher parent–infant attachment security, as observed with the abbreviated version of the AQS, was related to higher degrees of EE at the age of 16 years. This association was contrary to our expectations. In contrast, the serial mediation model with S-AQS and EE at the age of 16 years was also significant, and these results were in line with our hypothesis (i.e., lower parent–infant attachment security predicting more EE through increased suppression of emotions and alexithymia). Moreover, this mediational model explained more variance in EE than the model without the mediators (36% versus 29%, respectively). In addition, the results of the mediational model of S-AQS and EE at the age of 16 years were comparable to the findings with the S-AQS and SSSP at the age of 12 years, and the SSSP at the age of 16 years. In sum, while our findings seem to provide evidence for our initial hypothesis, future research is urged to investigate whether our results are robust and replicate.

Both attachment security measured with the SSSP and the S-AQS predicted EE in adolescence through increased suppression of emotions and alexithymia. This is an interesting finding, especially as these two measures of attachment were only moderately correlated (see Table 1). As such, these findings confirm the assumption that both attachment measures have predictive power of later child outcomes, but at the same time provide different windows into the parent–infant attachment relationship. While the SSSP reflects more the infant’s expectations of parental responsiveness in times of stress, the S-AQS reflects more the infant’s expectations of parental guidance in times of no stress [30]. As such, both attachment measures may therefore complement one another in predicting child outcomes, and infant expectations of parental responses in both times of stress and non-stress seem to be relevant for the development of EE.

This reflection on these attachment measures might also serve as an explanation for the results we found after including parental caregiving quality as a variable in the mediation models. In an earlier study on the same cohort, lower parental caregiving quality in infancy forecasted more EE in adolescence [40]. For this reason, the models were reconducted to investigate whether infant attachment security can predict EE above and beyond parental caregiving quality. While the models with the SSSP remained significant, the models with the S-AQS became insignificant. Parental caregiving quality and the S-AQS were both observed during non-stressful home observations, in contrast to the SSSP, which was observed during a stressful situation in the lab. So, compared to the SSSP, it could be hypothesized that the S-AQS and parental caregiving quality tap more into a similar part of the parent–infant interaction. However, strikingly, post-hoc correlations reveal that the association between the S-AQS and parental caregiving quality was less strong (*r* = 0.274, *p* = 0.006), compared to the association between parental caregiving quality and the SSSP (*r* = 0.421, *p* < 0.001). As such, these correlations do not seem to support our hypothesis. For future research, with a larger sample size compared to ours, it would be interesting to investigate to what extent attachment security and parental caregiving quality function as independent predictors of EE, and to what extent attachment security mediates the association between parental caregiving quality and EE. This knowledge would help to unravel the parts of the early parent–infant interaction relevant for the development of EE and inform programs for preventing EE in adolescence.

This study has many strengths. A first strength is the longitudinal, prospective nature of the study, following parents and their children over a time span of 15 years. Another strength is the multi-method approach used, including parent–infant attachment observations and child self-report to identify emotion suppression, alexithymia, and EE. A further strength is that parent–infant attachment security was observed with not one, but two measures: the SSSP and the S-AQS. The fact that the results were found to be similar with two different attachment measures gives confidence in the robustness of the results. We do have to note that it remains possible that the abbreviated versions of these attachment measures have negatively affected its validity. As a consequence, we can only wonder whether the predictive effects would have been larger when the unabbreviated versions would have been used.

We also have some other limitations to note. Suppression of emotions and alexithymia were measured at the same point in time, i.e., at the age of 12 years. As such, a reverse chain cannot be ruled out, but seems less likely given the literature to date (e.g., [43,44]. Nevertheless, for future studies, we recommend to measure suppression of emotions and alexithymia at different time points, so that more light can be shed on the direction of the chain. Another limitation is the relatively low sample size. Note that the bootstrapping approach was adopted to test the mediation models, as this approach yield results that are less influenced by sample size [58,59]. Nevertheless, the low sample size hampered power and precluded the possibility to investigate the possible moderating effect of child sex. Considering the higher degrees of EE in the female adolescents at the age of 16 years, it would be of interest to investigate possible sex differences in the serial mediation between attachment and EE. The low sample size also prevented us to investigate the different attachment classifications. As a meta-analysis in adults indicated that attachment anxiety was more strongly associated with unhealthy eating behaviors compared to attachment avoidance [13], it is possible that the four infant attachment classifications differently predict EE in adolescence. In addition, as the different dimensions of attachment security were shown to be related to distinct strategies of emotion regulation in adults [66,67], future studies should also investigate the different attachment classifications in relation to other child emotion regulation strategies, including cognitive reappraisal.

## 5. Conclusions

To the best of our knowledge, this study provides the first evidence that infant attachment security predicts adolescent EE longitudinally through mediation of emotion suppression and alexithymia. In line with studies showing associations between EE and obesity, and between attachment security and obesity [68,69,70], our findings also suggest that attachment security may contribute to healthier weights through healthier eating. Consequently, helping parents to improve their quality of parenting and the attachment security of their infants, may be an effective intervention to prevent EE and support healthier lifetime trajectories.

## Figures and Tables

**Table 1:** Correlations and descriptive statistics of the study variables at 12 (shown without brackets) and 16 years of age (shown between brackets).

	Attachment Security (SSSP)	Attachment Security (S-AQS)	Suppression of Emotions	Alexithymia	Emotional Eating	Child Sex	Parental Educational Level
Attachment security (SSSP)	–						
Attachment security (S-AQS)	0.27 ** (0.20)	–					
Suppression of emotions	−0.30 ** (−0.25 *)	−0.26 ** (−0.20)	–				
Alexithymia	0.04 (0.01)	−0.14 (−0.17)	0.33 ** (0.37 ***)	–			
Emotional eating	0.10 (0.14)	0.07 (0.28 **)	0.12 (−0.14)	0.44 *** (0.21 *)	–		
Child sex	0.15 (0.12)	0.15 (0.16)	−0.08 (−0.08)	0.18 (0.18)	0.15 (0.41 ***)	–	
Parental educational level	0.06 (−0.00)	0.01 (−0.06)	−0.08 (0.00)	−0.03 (−0.06)	0.14 (0.21 *)	−0.12 (−0.14)	–
M	0.6 (0.7)	0.3 (0.3)	3.3 (3.2)	9.4 (9.4)	1.7 (2.2)	1.5 (1.5)	5.2 (5.2)
Standard Deviation	0.5 (0.5)	0.3 (0.2)	1.2 (1.2)	2.3 (2.3)	0.7 (1.0)	0.5 (0.5)	1.6 (1.6)

Notes. *** *p* < 0.001; ** *p* < 0.01; * *p* < 0.05. S-AQS = shortened attachment Q sort; SSSP = shortened strange situation procedure.

## Data Availability

Data available on request from the authors.
